# Rare case of simultaneous cerebral artery and venous sinus thrombosis in the setting of elevated factor VIII and combined oral contraceptive pills

**DOI:** 10.1002/ccr3.5560

**Published:** 2022-03-15

**Authors:** Abeer Sabry Safan, Omnia A Hamid, Abdulrahman Al‐Mashdali, Arwa AlSaud, Shaikha Al‐Shokri, Abdelrahman Hamad

**Affiliations:** ^1^ Department of Neurology Neurosciences Institute Hamad Medical Corporation Doha Qatar; ^2^ 36977 Department of Internal Medicine Hamad Medical Corporation Doha Qatar

**Keywords:** cerebral venous sinus thrombosis, factor VIII, ischemic stroke

## Abstract

We report a rare case of combined cerebral venous sinus thrombosis and ischemic stroke in a 35‐year‐old female on combined oral contraceptive pills (COCPs) with persistently elevated factor VIII, presenting with headache and sudden onset vertigo, found to have extensive cerebral venous sinus thrombosis and PICA territory ischemic infarct.

## Graphical abstract

Due to the paucity of research to understand the pathophysiology of such rare occurrence, physicians must have a low threshold to explore factor VIII levels as part of their thrombophilia workup, possibly contributing to arterial and venous thrombosis.

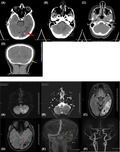

## 1 INTRODUCTION

Cerebral venous sinus thrombosis (CVST) is a cerebrovascular disease characterized by intercanal venous sinus occlusion that accounts for approximately 0.5%–1% of strokes.^1^ It has a peak incidence in middle‐aged individuals in their third decade of life of 3:1 female to male ratio.^2^ Compared to the sudden occurrence of neurologic deficits in arterial ischemic strokes, CVST may progress indolently over days relative to the location and extent of involved venous sinuses with a broad spectrum of clinical presentations. According to the most extensive cohort study on CVST, the International Study on Cerebral Venous and Dural Sinuses Thrombosis (ISCVT), 37% of patients present with acute onset (<48 h), 56% as subacute (>48 h to 30 days), and chronic (>30 days) in 7% of patients.^2^ CVST common risk factors encompass inherited and acquired prothrombotic conditions like hormonal therapy, malignancy, infection, and trauma‐related injuries.

Simultaneous cerebral arterial and venous infarction has been reported in very few case reports with homocysteinemia and acquired thrombophilia.^3^, ^4^, ^5^, ^6^ To the best of our knowledge, this is the first case of concurrent CVST and PICA ischemic stroke in a young female patient on COCP found to have elevated factor VIII.

## 2 CASE PRESENTATION

A 35‐year‐old Filipino female with no known past medical history presented to our hospital with a 5‐day history of severe occipital headache and sudden onset vertigo. Vertigo is the progressive rotatory sensation with no hearing loss or positional element associated with nausea, vomiting, and memory disturbance with alexia without agraphia. Further history revealed no fever, constitutional symptoms, travel, or recent sick contacts. There was no prior history of trauma or neck manipulation. She was on combined oral contraceptive pills (COCPs) for 3 months prior to admission. Family and past medical history were unremarkable for coagulopathy or strokes. Obstetric history revealed three previous uneventful pregnancies of normal vaginal delivery and no previous miscarriages or venous thromboembolic events (VTEs).

**FIGURE 1:**
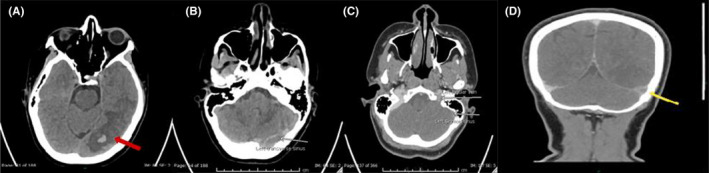
(A‐D) Plain‐ none enhanced CT head and CTV. (A, B) Left temporo‐occipital intraparenchymal hemorrhage with surrounding edema and adjacent mass effect evident by effacement of the adjacent cortical sulci. A cord like hypodensity noted in the left transverse sinus [white arrow]. (C, D) Filling defect involving the left transverse (yellow arrow) and left sigmoid sinuses extending to the left jugular vein. Left vein of Labbe is also not visualized [white arrows]

Physical examination revealed a conscious, alert, and oriented female with difficulty reading what she wrote consistent with alexia. Cranial nerve examination revealed right homonymous hemianopia with saccadic eye movements with right gaze‐evoked horizontal, non fatigable nystagmus on the right associated with right upper limb appendicular ataxia with truncal ataxia. The neck was supple and no other relevant finding. Laboratory investigations showed average complete cell counts. Vasculitis screen, including antiphospholipid syndrome, was negative, and complements levels (C3 and C4) were normal. Thrombophilia screen revealed normal protein C and S levels. Flow cytometry for paroxysmal nocturnal hemoglobinuria (PNH) was negative. Factor V Leiden mutation was absent, but a high level of factor VIII (243% reference 70%–150%) repeated factor VIII level within 10 days from the first level was 231.7%. Computed tomography venogram and angiogram (CTV and CTA) showed CVST with hemorrhagic venous infarct and vasogenic edema in the left temporal‐occipital with co‐current left vertebral occlusion with left PICA territory infarct (Figure 1).

**FIGURE 2:**
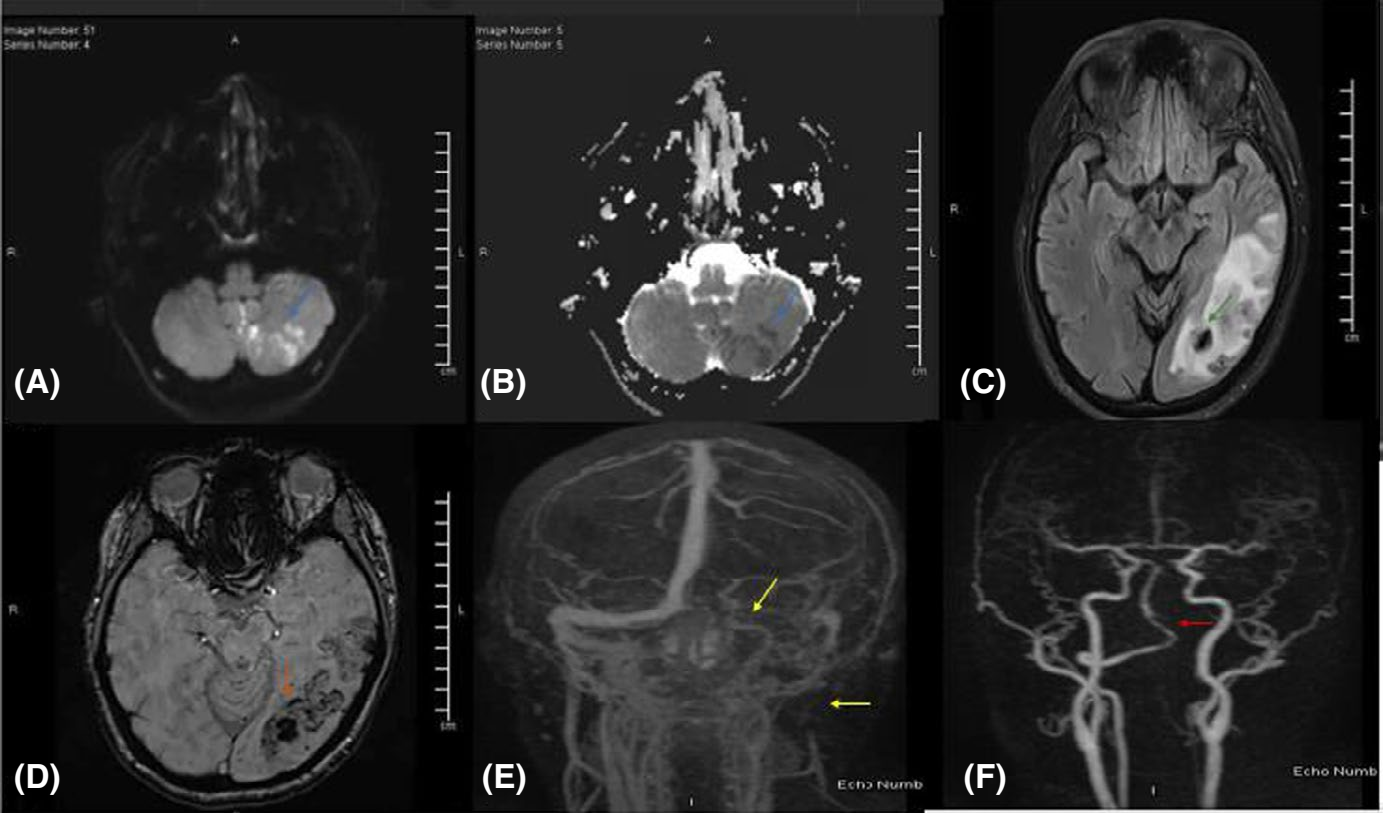
(A‐F) MRI/MRV/MRA head. (A, B) There is diffusion restricting (Blue arrow) and slightly FLAIR hyperintense acute infarcts in the left postero‐inferior cerebellar hemisphere (Green arrow). (C, D) SWI shows blooming in the left temporo‐occipital suggestive of venous hypertension with hemorrhage, with surrounding edema evident on FLAIR hyperintense (Orange arrow). (E, F) MRA, show occlusion of intracranial V4 and skull base V3 segments of the left vertebral artery (Red arrow). MR venogram shows that there is interval near total recanalization of the left sigmoid sinus, partial recanalization of the left internal jugular vein and significant amount of residual clot in the left transverse sinus medially with recanalization of only its lateral aspect (Yellow arrows)

Magnetic resonance image (MRI) and magnetic resonance venogram (MRV) within 24 h confirmed simultaneous venous thrombosis involving the vein of Labbe, left transverse, and left sigmoid sinuses extending to the left jugular vein with venous hemorrhage in the left temporo‐occipital territory along with acute left vertebral artery occlusion with left PICA territory infarct involving the cerebellar vermis with no features to suggest atherosclerosis (Figure 2A‐F). Echocardiogram was normal. Hence, stroke etiology is labeled as per Trial of ORG 10172 in acute stroke treatment (TOAST) classification as a stroke of undetermined etiology.^7^

The patient was started on therapeutic low molecular weight heparin (LMWH) enoxaparin (1 mg/kg subcutaneous twice per day) for 24 h, then switched to Dabigatran 150 mg twice per day for 3 months. COCP was discontinued, and further counseling of other contraceptive methods was conducted. A 2‐week follow‐up CT scan showed almost complete resolution of the hemorrhagic venous infarct. She was transferred to our rehabilitation institute for focused speech therapy, physical, and occupational therapy, for which her ataxia and stability improved significantly throughout the 8‐week program. She was discharged in good condition on anticoagulation with speech therapy sessions and outpatient stroke clinic follow‐ups.

A Three‐month follow‐up MRV showed complete recanalized venous sinuses, after which anticoagulation was stopped and was started on Aspirin 100 mg and continued with Atorvastatin 20 mg as secondary ischemic arterial stroke prevention.

## 3 DISCUSSION

Cerebral venous sinus thrombosis holds a higher rate of incidence among females, with 70%–80% of cases being females in their childbearing age.^2^ This disproportion can be explained by the most common etiology of CVST, attributed to hypercoagulable states, commonly in pregnancy, puerperium, and combined oral contraceptive use.^2^ Proposed mechanisms of CVST encompass thrombosis of cerebral veins or dural sinus impeding blood drainage leading to parenchymal insult and disruption of the blood–brain barrier, secondary to build up of venous and capillary pressure that impairs cerebral blood flow.^8^ Venous hemorrhagic infarcts ought to happen in nonresolved venous thrombus impairing cerebrospinal fluid (CSF) absorption via arachnoid granulation and increase intracranial pressure, leading to cytotoxic and vasogenic edema and eventually may cause parenchymal hemorrhage.^8^

Venous thromboembolism is a sequela of risk factors that trigger the Virchow triad (blood stasis, hypercoagulability, and vessel wall alteration). Risk factors of CVST are further classified as primary as in inherited coagulopathy or secondary to a parameningeal infection, trauma, neurosurgical procedures, and many inflammatory diseases that may co‐exist with a synergistic effect. For instance, combined oral contraceptive (COC) with co‐current mild to moderate inherited thrombophilia such as prothrombin gene mutation increases the odds of CVST in multiple folds. Mutated Factor V Leiden (R506Q) and MTHFR C677T are the most common genetically predisposed thrombophilia, accounting for 10% and 9.3%, respectively, of venous thromboembolism.^9^

Speaking of the coagulation cascade, factor VIII is a pivotal propagating factor of both intrinsic and extrinsic coagulation pathways that is stabilized by the von Willebrand factor complex (vWF); it has been linked to thrombotic states.^10^ Kreidy et al^9^. demonstrated a 5‐fold increased risk of thromboembolism and recurrence with factor VIII serum levels >150 mcg/L. Factor VIII level can be high in the acute phase of strokes, as O'Donnell et al. demonstrated in half of his patients showed persistently elevated factor VIII, which could support the notion that elevated factor VIII could be a driving force behind arterial thrombotic events.^10^

Factor VIII relevance in prothrombic syndrome was first recognized in 1990 when hemophiliac patients with factor VIII deficiency had a lower risk for coronary heart diseases.^11^ Over the past few years, compelling evidence showed the risk of arterial thrombosis linked with elevated serum factor VIII. Folsom et al. conducted a prospective study over 15,000 patients without cardiovascular risk factors who reported 191 ischemic strokes events that had factor VIII in the uppermost quartile showed an adjusted relative risk factor of 1.93.^12^ Our patient presented with a rare combination of veno‐arterial disease (ischemic stroke with CSVT), which had elevated factor VIII of 231.7% and 243.5% with the possible synergistic effect of COCP use to such rare clinical occurrence. Co‐current use of COCP and underlying thrombophilia can cause a coagulopathy synergistic effect and increase VTE risk. COCP is not only a risk factor for venous thromboembolic events but also for arterial strokes, with the world health organization (WHO) estimating an odds ratio of 2.99 (1.65–5.40).^1^ In a systematic review and meta‐analysis on COCP, thrombophilia and the risk of venous thromboembolism showed a 6‐fold increased risk of VTE in COCP users with mild to severe thrombophilia with a rate ratio [RR], 5.89; 95% confidence interval [CI], (4.21–8.23).^10^ Cochrane reviews noted that the use of combined oral contraceptives increased the risk of venous thrombosis (relative risk (RR) 3.5, 95% confidence interval (CI) 2.9–4.3) and arterial thrombosis (RR1.7, 95% CI 1.5–1.9) compared with nonusers.^13^

Simultaneous CVST and ischemic arterial infarcts have been reported before in the setting of profound iron deficiency anemia, thrombocytosis, hyperhomocysteinemia, and in patients with SARS‐CoV‐2 (COVID‐19) infection.^3^, ^4^, ^6^ Bo‐Lin Ho et al^5^. reported a case of simultaneous CSVT and ischemic stroke in 41 years old with acquired protein C and protein S deficiency, iron deficiency anemia (IDA), and cryoglobulinemia, which were within normal in our patient. Relative to our patient and the best of our knowledge, none of these cases had reported elevated factor VIII.

## 4 CONCLUSION

Simultaneous arterial and venous thrombosis is a rare occurrence, where exploring predisposing risk factors and extensive stroke workup of primary and secondary causes is warranted. Synergistic thromboembolic factors increase the odds of presenting with possible multiple undiscovered pathophysiologies. Our patient had 1.6‐fold higher serum factor VIII with concurrent use of COCP for 3 months, which we believe might have contributed to her rare presentation in the setting of the unrevealing workup of stroke in the young. Further studies are warranted to understand the role of factor VIII in arterial and venous thromboembolic events.

## CONFLICT OF INTEREST

The authors have no conflict of interest to declare.

## AUTHOR CONTRIBUTIONS

AS, OH, AM, AA, and SS involved in writing the initial draft of the manuscript. SS and AAZ involved in conceptualization and supervision. AS, OH, AM, AA, SS, and AAZ involved in medical management of the case. AS, OH, AM, AA, SS, and AA involved in revising the manuscript critically and literature review.

## ETHICAL APPROVAL

This case report was approved by the Hamad Medical Corporation's Medical Research Center (Protocol number: MRC‐04‐21‐575).

## CONSENT

Written informed consent was obtained from the patient for the publication of this case report.

## Data Availability

The datasets used and/or analyzed during the current study are available from the corresponding author on request.
